# The dark side of algorithmic entertainment: social and physical presence, short video addiction, and cognitive fatigue among Douyin users

**DOI:** 10.3389/fpsyg.2026.1856148

**Published:** 2026-06-15

**Authors:** Kaige Bai, Yuliang Liu

**Affiliations:** 1 School of Media and Communication, Taylor’s University, Subang Jaya, Selangor, Malaysia; 2 Hebei Academy of Fine Arts, Shijiazhuang, Hebei Province, China

**Keywords:** cognitive fatigue, fsQCA, hedonic pleasure, loss of control, physical presence, PLS-SEM, short video addiction, social presence

## Abstract

Short video platforms such as Douyin have become deeply embedded in everyday digital life, reshaping leisure, emotion, and identity, but their immersive features also pose cognitive and psychological risks. This study examines how social presence and physical presence jointly influence two interrelated outcomes among young Chinese users: short video addiction and cognitive fatigue. Using survey data from 649 participants, we employed both PLS-SEM and fsQCA to identify linear relationships and configurational patterns. Results show that all hypothesized relationships are supported except the direct effect of emotional fatigue on cognitive fatigue. Social presence primarily increases addiction through hedonic pleasure, whereas physical presence exerts its influence through loss of control. The fsQCA findings further reveal that severe cognitive deterioration emerges not from any single factor but from dynamic combinations of emotional, cognitive, and behavioral conditions. Integrating presence and media theories, this study links algorithmic engagement to mental health risks. Given the cross-sectional design, the findings should be interpreted as associative rather than causal.

## 1 Introduction

Short videos on Douyin have demonstrated unique advantages over traditional media by reshaping user–content interaction and the overall media experience (Wang et al., 2024; Kaye et al., 2021). Especially in recent years, short-form video platforms have increasingly been recognized as a distinct digital environment with unique psychological and cognitive implications (Kruzan and Won, 2019; Siles et al., 2024). Several recent high-quality systematic reviews and meta-analyses (2025–2026) further confirm that short-video use is significantly associated with mental health problems, addictive behaviors, and cognitive impairments (Nguyen et al., 2025; Tang et al., 2026; Mona et al., 2026; Li et al., 2025). These findings provide strong and direct evidence for understanding the psychological and cognitive consequences of short-video consumption.

However, this transformation is not purely positive. While short videos generate immersion and immediacy, they also introduce distraction, addictive tendencies, and cognitive biases, highlighting their dual nature as a “double-edged sword” (Chen et al., 2023; Zhou et al., 2023; Qu et al., 2024).

First, Douyin’s rich Interaction Features (IF)—such as likes, comments, shares, and real-time feedback—create a socially interactive ecology that enhances user engagement and perceived participation (Kim et al., 2021; Pang, 2021). Second, diverse content enables Emotional Release (ER), allowing users to regulate emotions through humorous or affective narratives, thereby providing psychological comfort and stress relief (Thomas, 2022; Ning et al., 2024; Rimé et al., 2020). Third, through narrative immersion and audiovisual stimulation, short-video platforms foster strong Role Immersion (RI), encouraging users to psychologically engage with virtual roles and storylines, thereby enhancing presence and influencing subsequent attitudes and behaviors (Cadet and Chainay, 2020; Daassi and Debbabi, 2021).

Moreover, Douyin’s Precision Algorithmic Recommendation (PAR) system tailors content to individual preferences, increasing engagement and perceived relevance (Meng, 2021; Zhang et al., 2020; Siles et al., 2024). Combined with Hedonic Pleasure (HP), such personalized and entertaining content provides instant gratification and facilitates emotional regulation under daily stress (Reybrouck and Eerola, 2022; Pang, 2021). However, these strengths also introduce potential cognitive and behavioral risks.

Recent evidence indicates that the fragmented and fast-paced nature of short-video content is associated with attentional deficits and impaired cognitive processing (Mona et al., 2026). Specifically, Fragmented Information (FI)—characterized by brief, discontinuous, and rapidly shifting content—can disrupt deep information processing and critical thinking (Rosen et al., 2013; Saker and Frith, 2020). Continuous content switching further contributes to Attention Deprivation (AD), weakening sustained focus and increasing cognitive overload (Rosen et al., 2013; Chen et al., 2023). In addition, algorithm-driven recommendation mechanisms reinforce repetitive viewing patterns, increasing the likelihood of Loss of Control (LC) over time management and usage behavior (Gilbert et al., 2024; Jiang and Yoo, 2024). Over time, such patterns may lead to Short-Video Addiction (SVA), which is associated with psychological distress, including anxiety, depression, and social withdrawal (Egliston and Carter, 2024; Qu et al., 2024).

Consistent with recent meta-analytic findings, excessive short-video use is strongly associated with psychological distress, including anxiety and depression (Nguyen et al., 2025; Tang et al., 2026). Prolonged exposure may also induce Emotional Fatigue (EF), reducing users’ enjoyment and increasing emotional exhaustion (Sheng et al., 2023; Hsu et al., 2024; Kaur et al., 2021). Meanwhile, Time Distortion (TD) can lead users to misperceive usage duration and neglect offline responsibilities. Reduced real-world interaction may further result in Reality Social Avoidance (RSA), weakening interpersonal relationships and increasing perceived loneliness (Miguel et al., 2024; Kruzan and Won, 2019). Ultimately, persistent exposure to simplified and fragmented content contributes to Cognitive Fatigue (CF), manifested as declines in concentration, memory, and decision-making capacity (Faraci and Nasonte, 2024; Hsu et al., 2024).

Despite the growing body of systematic evidence, several limitations remain in existing research. First, prior studies predominantly focus on usage motivations or behavioral patterns, while overlooking cognitive consequences such as AD and CF. Second, Social Presence (SP) and Physical Presence (PP)—core constructs for understanding immersive media experiences—have been extensively examined in virtual reality and gaming contexts but remain underexplored in short-video environments (Cadet and Chainay, 2020; Daassi and Debbabi, 2021; Yildiz et al., 2024). Third, although recent reviews have identified addiction and mental health outcomes, there is still a lack of an integrated framework that connects presence-related constructs, behavioral mechanisms, and cognitive consequences within short-video contexts. Finally, from a methodological perspective, most existing studies rely on symmetrical approaches such as regression or structural equation modeling, which primarily capture net effects but overlook complex causal configurations.

Therefore, this study proposes four research objectives. The first objective (RO1) is to examine how SP (operationalized through IF, ER, and RI) and PP (operationalized through FI, PAR, and AD) influence SVA and CF. The second objective (RO2) is to investigate the mediating role of HP in the relationship between SP and SVA, as well as the mediating role of LC in the relationship between PP and SVA. The third objective (RO3) is to explore how EF, TD, and RSA shape the relationship between SVA and CF. The fourth objective (RO4) employs a complementary analytical design combining Partial Least Squares Structural Equation Modeling (PLS-SEM) and fuzzy-set Qualitative Comparative Analysis (fsQCA). Specifically, PLS-SEM is used to test causal and mediating relationships (RO1–RO3), while fsQCA identifies multiple configurations of conditions leading to SVA and CF (RO4). This integrated approach enhances analytical robustness and provides a more comprehensive understanding of short-video consumption behavior, offering both theoretical and methodological contributions.

## 2 Literature review

Although previous studies have highlighted the unique advantages of short videos in terms of interactivity, immersion, and content dissemination, significant gaps remain in theoretical construction and the integration of key variables (Wang, 2020; Wang et al., 2024; Siles et al., 2024). This study adopts four complementary theoretical perspectives to develop a systematic analytical framework. Specifically, presence theory explains how algorithm-driven environments generate immersive experiences (Cadet and Chainay, 2020; Daassi and Debbabi, 2021; Yildiz et al., 2024), Gratifications Theory (UGT) accounts for hedonic responses (Pang, 2021; Reybrouck and Eerola, 2022), media dependence theory explains the emergence of behavioral dependence (Meng, 2021; Zhang et al., 2020), and information overload theory captures the resulting cognitive consequences (Kaur et al., 2021; Sheng et al., 2023). Together, these perspectives form a coherent mechanism linking algorithmic engagement to cognitive fatigue (Hsu et al., 2024; Faraci and Nasonte, 2024). Importantly, these theories are not applied in parallel but are conceptually embedded within a unified process: presence theory provides the experiential foundation (Cadet and Chainay, 2020), UGT explains immediate affective responses (Pang, 2021), media dependence theory captures behavioral reinforcement (Meng, 2021), and information overload theory explains cognitive consequences (Sheng et al., 2023; Hsu et al., 2024). This layered integration allows the model to explain not only what happens, but how and why these processes unfold sequentially. This study is primarily grounded in presence theory as the central explanatory lens, with UGT, media dependence theory, and information overload theory serving as complementary mechanisms that explain subsequent psychological and behavioral processes.

First, research on SP and PP has largely focused on virtual reality and immersive media environments (Cadet and Chainay, 2020; Daassi and Debbabi, 2021; Yildiz et al., 2024), while the complex mechanisms linking IF, information fragmentation, algorithmic precision, and AD in the context of short videos remain underexplored (Siles et al., 2024; Zhang et al., 2020). Second, although media dependence theory can explain users’ structural reliance on media, it provides limited insight into the dynamic process by which users’ LC evolves into SVA (Meng, 2021; Qu et al., 2024). Third, although UGT has been applied to explain entertainment and informational motivations in short-video use (Pang, 2021; Wang, 2020), its capacity to capture deeper psychological pathways such as ER, RI, and hedonic satisfaction is still insufficient (Rimé et al., 2020; Reybrouck and Eerola, 2022). Finally, information overload theory offers a robust perspective for understanding the negative consequences of digital media (Kaur et al., 2021; Sheng et al., 2023), yet its explanatory power in short-video contexts—particularly regarding EF, TD, RSA, and CF—requires further validation (Hsu et al., 2024; Faraci and Nasonte, 2024; Miguel et al., 2024).

In summary, these four theories address short-video use from four complementary dimensions—presence, dependence, psychological motivation, and information load—collectively forming a framework for understanding the “double-edged sword” effect of short videos (Chen et al., 2023; Zhou et al., 2023; Qu et al., 2024). Building on this, the present study aims to integrate these theoretical perspectives to systematically examine the interactions and mechanisms among these variables, providing a more comprehensive and nuanced explanation of short-video usage.

### 2.1 SP in short videos

SP has become a core element of the Douyin viewing experience. Studies show that IF such as commenting, liking, sharing, and real-time engagement influence user behavior and content circulation (Jabeen et al., 2023; Yildiz et al., 2024). Beyond content dissemination, interaction fosters emotional resonance and virtual connections, strengthening users’ sense of belonging. Short videos also enable ER, offering stress relief (Fontes-Perryman & Spina, 2022; Saker and Frith, 2020), and provide RI, where users temporarily embody different identities.

Existing studies treat interaction, ER, and RI separately, rarely examining how they jointly construct SP. The lack of integration leaves unanswered how these processes collectively enhance belonging and community attachment.

Theoretically, neglecting the mediating role of SP weakens explanations of emotional regulation and immersion. Practically, platforms that emphasize only interaction or entertainment may overlook deeper social and emotional needs vital for user well-being and sustainable engagement.

This study analyzes these mechanisms as interrelated contributors to SP, clarifying its role in linking user interaction, emotion, and immersion. Yet, IF alone are insufficient; the physical presentation of short videos must also be considered. In contrast to PP, which is driven by algorithmic content structuring, SP reflects socially constructed immersion, highlighting two distinct but complementary forms of presence in short-video environments.

### 2.2 PP in short videos

Unlike traditional VR-based research, where physical presence primarily refers to spatial immersion and the sensation of “being there” within a virtual environment (Cadet and Chainay, 2020; Daassi and Debbabi, 2021), short-video platforms operate through two-dimensional, algorithm-driven interfaces rather than immersive three-dimensional spaces (Saker and Frith, 2020). Therefore, PP in short-video contexts should not be understood as literal spatial embedding. Instead, this study conceptualizes PP as an algorithmically mediated perceptual immersion that emerges through continuous interaction with dynamically structured content flows (Meng, 2021; Siles et al., 2024).

Specifically, FI structures rapid and fragmented perceptual flow (Rosen et al., 2013; Saker and Frith, 2020), PAR enhances perceived relevance and continuity of content exposure (Zhang et al., 2020; Siles et al., 2024), and AD captures and sustains attentional engagement during continuous viewing (Chen et al., 2023; Rosen et al., 2013).

Together, these mechanisms create a persistent sense of “being immersed in content flow,” generating immersion-like psychological experiences despite the absence of spatial immersion (Cadet and Chainay, 2020; Yildiz et al., 2024).

In algorithm-driven short-video environments, PAR increases perceived personalization and viewing continuity while simultaneously narrowing informational diversity, whereas rapid content turnover intensifies attentional capture and cognitive engagement (Siles et al., 2024; Miguel et al., 2024; Chen et al., 2023). Although FI, PAR, and AD have been widely examined in prior studies, they are typically investigated independently rather than as interrelated mechanisms shaping presence-related experiences (Zhang et al., 2020; Sheng et al., 2023). Consequently, how these algorithmic features jointly construct perceptual immersion and influence subsequent psychological outcomes remains insufficiently understood (Cadet and Chainay, 2020; Daassi and Debbabi, 2021).

Theoretically, this limitation constrains existing explanations of how media system characteristics shape users’ psychological states and behavioral outcomes (Meng, 2021; Pang, 2021). Practically, platform designs that prioritize engagement efficiency without considering attentional consequences may contribute to reduced attentional control, compulsive consumption patterns, and restricted informational exposure (Chen et al., 2023; Qu et al., 2024; Gilbert et al., 2024). Addressing this issue is therefore important for both theoretical development and user well-being.

Accordingly, this study reframes FI, PAR, and AD as interrelated components contributing to PP within short-video environments. Importantly, this conceptualization does not attempt to equate short-video immersion with VR-based spatial presence. Rather, it extends presence theory from spatial immersion contexts to algorithmically mediated perceptual immersion in contemporary short-video platforms (Daassi and Debbabi, 2021; Yildiz et al., 2024). In this context, the sense of “being in” the media environment emerges from sustained perceptual engagement, attentional capture, and continuous content interaction rather than from spatial simulation or virtual embodiment (Siles et al., 2024; Meng, 2021). Through these mechanisms, users experience an ongoing sense of immersion in algorithmically curated content flow, which constitutes PP in short-video contexts. In conclusion, This study therefore conceptualizes PP as a contextual extension of presence theory rather than a direct replication of VR-based physical presence.

### 2.3 Media dependence theory

On platforms like Douyin, rapid updates and precise recommendations often trap users in “endless scrolling,” undermining control over time and content (Zhang et al., 2022; Peterson-Salahuddin, 2024). Research shows that algorithmic precision and constant novelty foster a state of information LC (Liu et al., 2021; Zhang et al., 2024). Within media dependence theory, such compulsive use can evolve into over-dependence, marked by prolonged viewing, heightened demand, and offline withdrawal (Weber et al., 2020). Instant feedback and fragmented, personalized content further trigger addictive behaviors (Wang et al., 2015).

Most studies describe behavioral outcomes (e.g., time spent) but overlook how LC connects with psychological needs and emotions. The role of psychological dependence is rarely integrated into models, and how LC and addiction intertwine with SP, emotional resonance, and immersion remains unclear.

This gap narrows theoretical explanations of how structural reliance becomes deeper dependence and limits practice, as governance strategies that target screen time alone ignore emotional vulnerabilities driving compulsive use—leaving risks of exhaustion, withdrawal, and CF unaddressed.

This study treats LC and addiction as psychological as well as behavioral processes within media dependence. By linking them to emotional and social mechanisms, it advances a fuller account of the short-video experience. Yet dependence also involves motivational gratifications—best examined through Uses and Gratifications Theory (UGT).

### 2.4 UGT

Within the UGT framework, users actively engage with media to meet diverse needs that shape usage patterns. In short videos, SP fosters emotional connection and interaction (Grall et al., 2021; Akbari and Pishghadam, 2022), while PP enhances immersion through FI, algorithmic precision, and AD. Entertainment satisfaction, a core UGT construct, stems from pleasure and relaxation, with platforms offering instant gratification and diverse content that reinforce dependence (Gilbert et al., 2024; Daassi and Debbabi, 2021; Sheng et al., 2023).

Research has mostly examined social interaction and ER, leaving RI and its link to SP underexplored. The combined effects of fragmented content, precision recommendations, and AD on immersion remain undertheorised. Similarly, the relationship between entertainment satisfaction and other needs (e.g., social connection, emotional resonance) is insufficiently analyzed. Finally, while SVA is documented, the psychological mechanism by which excessive satisfaction drives LC is unclear.

This limits UGT’s explanatory power: without integrating entertainment satisfaction, RI, and presence dynamics, theories risk overlooking how gratifications evolve into compulsive use. Practically, interventions targeting behavior alone (e.g., screen time) neglect deeper psychological gratifications that sustain addiction.

This study positions entertainment satisfaction, SP, and PP within UGT to explain how gratifications intertwine with dependence. It highlights how excessive need fulfillment fosters LC, extending UGT from motivations to maladaptive outcomes.

### 2.5 Information overload theory

Information overload theory explains the psychological strain individuals face when processing excessive information (Jackson & Farzaneh, 2012; Pernagallo & Torrisi, 2022). On short-video platforms, rapid updates, fragmented content, and intense social interactions amplify this overload, leading to EF (Li and Ma, 2024). Users also experience TD, as personalized recommendations and fast-switching content make them lose track of viewing duration (Yu, 2021; Kim et al., 2021). Prolonged immersion may trigger RSA, where users withdraw from offline interactions (Irom, 2022; Zhang et al., 2020), and CF, reflected in reduced attention, memory, and decision-making capacity. In this study, cognitive fatigue is conceptualized as a state of cognitive overload and resource depletion, rather than cognitive activation or enhancement.

**Figure 1:**
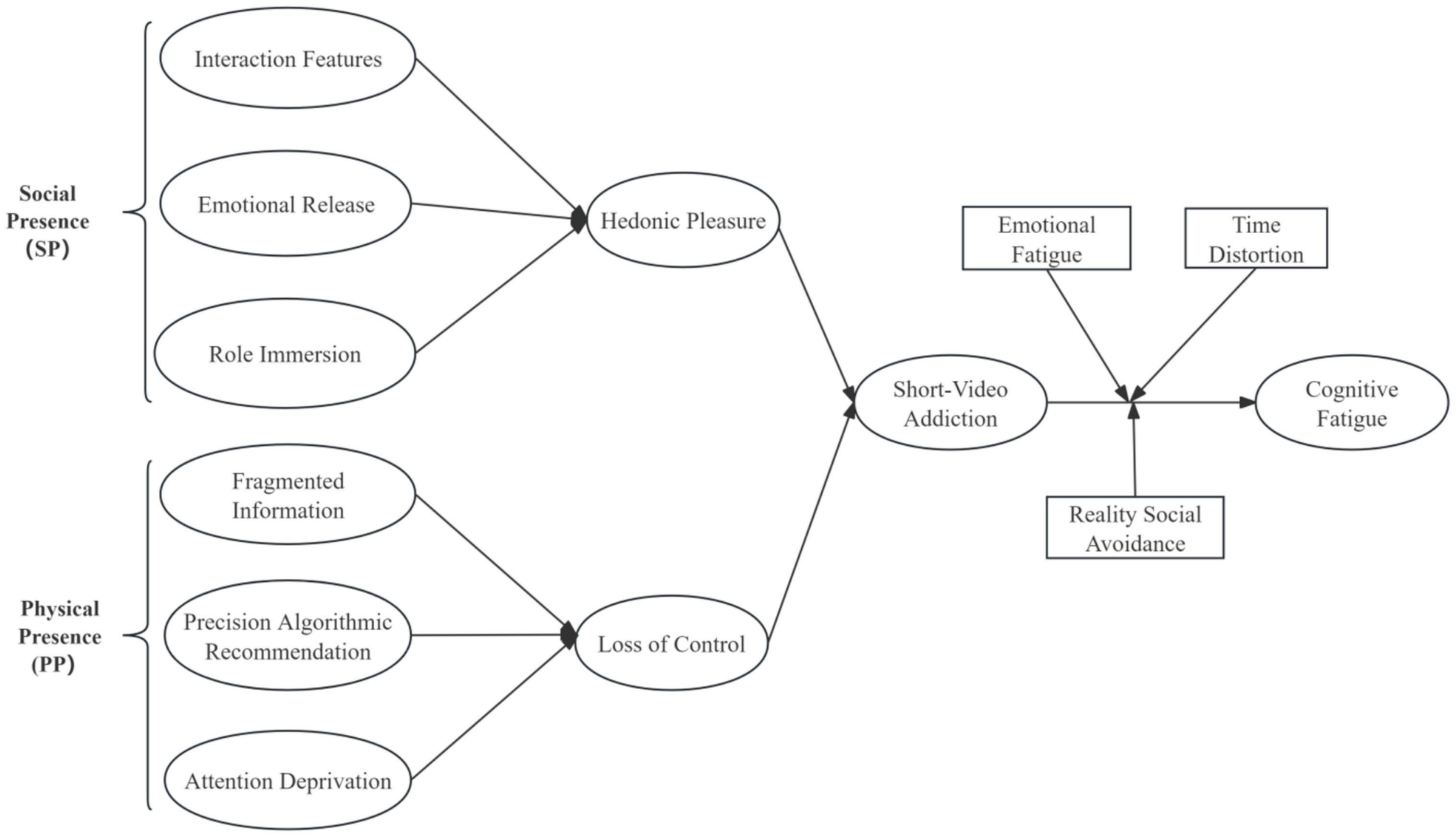
Conceptual framework. The model illustrates two core pathways: a hedonic pathway (SP (IF, ER, and RI) → HP → SVA → CF) and a control-loss pathway (PP (FI, PAR, and AD) → LC → SVA → CF).

Overall, information overload theory provides a robust lens for examining how fragmented, high-volume content affects users’ psychological states and behaviors, forming the basis of this study’s conceptual framework (Figure 1). Although multiple constructs are included, the model maintains theoretical parsimony by organizing them into two core pathways—a hedonic pathway and a control-loss pathway—which together capture the core mechanisms through which presence translates into cognitive fatigue.

## 3 Hypothesis development

Building on the integrated theoretical framework established in Section 2, this study conceptualizes the effects of presence on cognitive fatigue through two core pathways: a hedonic pathway and a control-loss pathway. Rather than examining isolated relationships, the model emphasizes how SP and PP operate as overarching mechanisms that shape psychological responses and behavioral outcomes (Cadet and Chainay, 2020; Yildiz et al., 2024).

### 3.1 Hedonic pathway

As discussed in Section 2.1 and 2.4, SP represents socially constructed immersion that enhances interaction, emotional resonance, and role engagement (Kruzan and Won, 2019; Cadet and Chainay, 2020). Within SP, IF enable instant feedback through likes, comments, and sharing, stimulating cognitive engagement and information processing (Kim et al., 2021; Pang, 2021). ER allows users to release stress and maintain psychological balance (Rimé et al., 2020), while RI promotes identification and involvement with virtual roles, strengthening psychological immersion and satisfaction (Daassi and Debbabi, 2021; Yildiz et al., 2024). These experiences collectively generate HP by fulfilling users’ needs for enjoyment, emotional release, and social connection (Reybrouck and Eerola, 2022; Pang, 2021).

*H1*: SP positively influences HP.

According to UGT, sustained HP motivates repeated media use and reinforces engagement behavior. Users tend to seek pleasurable experiences derived from audiovisual stimulation and social interaction (Pang, 2021; Wang, 2020). Over time, the pursuit of pleasure is linked to habitual and compulsive viewing patterns, thereby increasing SVA (Hsu et al., 2024; Jiang and Yoo, 2024).

*H2*: HP positively influences SVA.

### 3.2 Control-loss pathway

As conceptualized in Section 2.2 and 2.3, PP reflects an algorithmically mediated perceptual experience shaped by FI, PAR, and AD (Meng, 2021; Siles et al., 2024). FI fragments attention and disrupts sustained cognitive processing (Rosen et al., 2013; Saker and Frith, 2020), while PAR continuously delivers personalized content that may reduce user autonomy and reinforce passive consumption patterns (Meng, 2021; Zhang et al., 2020; Qu et al., 2024). Meanwhile, frequent AD induced by rapid content turnover and multimodal stimuli depletes attentional resources and weakens concentration (Chen et al., 2023; Rosen et al., 2013). These features collectively reduce users’ ability to regulate their media consumption, leading to LC (Gilbert et al., 2024; Jiang and Yoo, 2024).

*H3*: PP positively influences LC.

From the perspective of media dependence theory, reduced self-regulation increases users’ reliance on the platform. Continuous exposure to algorithm-driven content gradually reinforces habitual engagement and leads to compulsive and excessive usage (Meng, 2021; Miguel et al., 2024; Qu et al., 2024).

*H4*: LC positively influences SVA.

### 3.3 Cognitive outcome

Drawing on information overload theory, prolonged and excessive engagement with short-video platforms consumes cognitive resources and impairs attention, memory, and information processing (Kaur et al., 2021; Sheng et al., 2023; Gilbert et al., 2024). SVA, characterized by persistent and uncontrolled usage, intensifies cognitive load and reduces users’ ability to recover, ultimately leading to CF (Faraci and Nasonte, 2024; Hsu et al., 2024).

*H5*: SVA positively influences CF.

### 3.4 Sequential mediation

Integrating the above pathways, SP and PP are expected to influence CF indirectly through sequential mediation processes involving HP, LC, and SVA. Specifically, SP enhances HP, which promotes SVA and ultimately contributes to CF, while PP increases LC, which strengthens SVA and subsequently leads to CF (Hsu et al., 2024; Jiang and Yoo, 2024; Faraci and Nasonte, 2024).

*H6*: HP and SVA jointly mediate the relationship between SP and CF.

*H7*: LC and SVA jointly mediate the relationship between PP and CF.

### 3.5 Moderating effects

Consistent with Research Objective 3, this study examines whether EF, TD, and RSA moderate the relationship between SVA and CF. EF may amplify CF by exhausting emotional and attentional resources (Sheng et al., 2023; Hsu et al., 2024), TD may distort temporal perception and reduce cognitive awareness (Gilbert et al., 2024), and RSA may weaken real-world social interaction and reduce cognitive stimulation (Miguel et al., 2024; Kruzan and Won, 2019). These factors may intensify or attenuate the cognitive consequences of SVA.

*H8*: EF, TD, and RSA moderate the relationship between SVA and CF.

## 4 Research methods

In the context of the increasing popularity of Douyin short-video viewing, the mechanisms that shape the CF and its potential negative influence must be explored (Kaur et al., 2021; Sheng et al., 2023). This study carefully adapted several scales derived from supported research to accommodate the uniqueness and complexity of the short-video communication process (Pang, 2021; Xu et al., 2024). These scales are clearly presented to participants as statements designed to accurately measure their attitudes. A five-point Likert scale, with one representing “strongly disagree” and five representing “strongly agree,” is created to quantify participants’ level of agreement to ensure that participants could fully express their opinions on each statement (Hair et al., 2017). Refer to the Appendix for additional information on the structure of the scale, specific items and sources. All measurement items were adapted from established scales (see Supplementary Table 1 for full item descriptions and sources).

This study focuses on users aged 18–35 because they constitute the main user base on Douyin and represent the demographic most prone to immersive and addictive short-video use (Jiang and Yoo, 2024; Qu et al., 2024). The term “CF” in this study does not refer to clinical age-related cognitive disorders but rather to reductions in cognitive functioning—such as attentional lapses and weakened memory—resulting from information overload, AD, and excessive use (Kaur et al., 2021; Faraci and Nasonte, 2024). Focusing on this young adult group aligns with the research objectives. Future studies may extend the investigation to middle-aged and older populations to compare the differential impacts across age groups.

We adopted a purposeful sampling method (Campbell et al., 2020) to select samples of people who use Douyin short videos. This approach is adopted because it allows the selection of respondents who meet specific criteria (Hair et al., 2017). The final questionnaire is released through www.credamo. The screening questions are set up, and people who are not able to use Douyin short videos and are under 18 years old and over 35 years old are excluded. The participants who complete the survey will receive a reward of about US$5. The study protocol was approved by an institutional research ethics committee. All participants provided informed consent prior to participation. This study adopts a cross-sectional design, which limits causal inference (Hair et al., 2017).

The sample size is determined using the G*Power tool to ensure the robustness of the statistical analysis and improve the reliability and validity of the research results, (Erdfelder et al., 2009; Faul et al., 2007). Considering the 13 predictive variables included in the model, the power level is 95%, and the significance level is 0.05 based on these two parameters, in accordance with the criterion proposed by Cohen (1988). At least 262 samples must be collected. The study found 649 valid responses far exceeding the minimum recommended sample size. In the analytical methods, symmetric techniques (SEM-PLS) and asymmetric techniques (fsQCA) are used to provide a comprehensive and detailed assessment of the assumptions of the research model (Hair et al., 2017; Fiss, 2011).

A total of 710 questionnaires were initially distributed. After quality screening, 61 invalid responses were removed (35 completed in less than 120 s, 18 with consecutive identical answers, and 8 with missing rates exceeding 10%), resulting in 649 valid responses (N = 649). The demographic characteristics of the sample are as follows: 301 males (46.4%) and 348 females (53.6%). Educational attainment was distributed as follows: high school or below 97 (15.0%), associate degree 73 (11.2%), bachelor’s degree 373 (57.5%), and master’s degree or above 106 (16.3%). Regarding Douyin usage, respondents reported an average daily usage of 1.9 h (SD = 1.2) and an average frequency of 4.8 times per day (SD = 2.6). Geographically, participants were distributed across China as follows: Eastern region 286 (44.1%), Central region 172 (26.5%), Western region 118 (18.2%), Northeast 39 (6.0%), and other regions 34 (5.2%).

During data preprocessing, questionnaires with completion times under 120 s, consecutive identical responses, or missing rates above 10% were removed (totaling 61). The remaining sample was checked for missing values, with an overall variable-level missing rate of approximately 0.9%. Listwise deletion was applied during modeling to ensure consistent estimation, resulting in a final effective sample of N = 649 for PLS-SEM analysis (a few records missing key control variables were removed) (Hair et al., 2017).

The measurement model demonstrated satisfactory reliability and validity. All factor loadings exceeded 0.70, composite reliability (CR) values were above 0.70, average variance extracted (AVE) values exceeded 0.50, and Cronbach’s alpha values were above 0.70, indicating good internal consistency and convergent validity (Hair et al., 2017). Full measurement results are presented in the Supplementary Table 2. Discriminant validity was assessed using the HTMT criterion, and all values were below the recommended threshold, suggesting that the constructs were empirically distinct. Although some constructs (e.g., SP-related and PP-related dimensions) are conceptually related, the results still supported adequate discriminant validity.

Common method bias (CMB) was assessed using multiple approaches. First, a full collinearity test was conducted, and all Variance Inflation Factor (VIF) values ranged from 1.576 to 2.187, well below the recommended threshold of 3.3, indicating no severe multicollinearity issues (Kock, 2015). Detailed VIF results are provided in the Supplementary Table 3. Second, Harman’s single-factor test showed that the first factor accounted for less than 50% of the total variance, suggesting that common method bias was not a serious concern (Kaur et al., 2021; Sheng et al., 2023). These results indicate that the risk of CMB was adequately controlled (Hair et al., 2017).

## 5 Results

### 5.1 Symmetric analysis

PLS-SEM, a symmetric technique, is particularly suitable for dealing with complex models and exploratory studies (Hair et al., 2017; Hair Jr. et al., 2016) and can reveal the relationships among variables between underlying variables. Although other symmetric techniques can assist researchers in identifying the key factors and significant patterns that is linked to a particular outcome, they have difficulty in capturing the complexity of the determinants that influence the outcome and their nonlinear dynamics (Dul, 2016). Therefore, other PLS methods and regression analyses are ruled out in this context.

#### 5.1.1 Measurement model evaluation

In the process of evaluating the measurement model, we calculated several key indicators, including ‘loadings’, ‘combined reliability’ (CR), ‘average variance extraction’ (AVE) and ‘discriminant validity.’ After calculation, the values of the index load, CR and AVE significantly exceed their preset reference thresholds of 0.7, 0.7 and 0.5, respectively (Supplementary Table 2 Measurement model assessment results).

This study used the heterogeneity–uniformity correlation ratio (HTMT) to determine discriminant validity. The study results showed that each HTMT value is below the recommended threshold of 0.9, confirming adequate discriminant validity (Supplementary Table 4 HTMT results).

#### 5.1.2 Structural model evaluation

This study tested the structural model by evaluating the explained variance (R-square), path coefficient (*β*) and its related t-value. The R2 value represents the proportion of the dependent variable’s variation that the independent variables can explain. Falk and Miller (1992) stated that an R2 value greater than 0.10 is considered substantial. The R-square value of the endogenous factors shows that our proposed model explains 34% of CF, 34% of HP, 28.4% of LC and 32.3% of SVA. The results indicated that all direct relationships are confirmed, except the effect of EF on CF. However, in the moderation effect, EF, TD and RSA have no moderating effect on the influence of SVA on CF (Table 1).

**Table 1:** Structural model results.

H	Construct	Path	*β*	*t*	*p*	Decision
H1	SP (IF)	IF → HP	0.312	6.479	0	Supported
	SP (ER)	ER → HP	0.191	4.049	0	Supported
	SP (RI)	RI → HP	0.227	5.044	0	Supported
H2		HP → SVA	0.29	7.273	0	Supported
H3	PP (FI)	FI → LC	0.193	4.808	0	Supported
	PP (PAR)	PAR → LC	0.261	6.337	0	Supported
	PP (AD)	AD → LC	0.234	5.474	0	Supported
H4		LC → SVA	0.378	9.816	0	Supported
H5		SVA → CF	0.18	3.965	0	Supported
H8	Moderation	EF × SVA → CF	0.03	0.72	0.471	Not supported
		RSA × SVA → CF	−0.002	0.039	0.969	Not supported
		TD × SVA → CF	0.014	0.296	0.767	Not supported

IF, Interaction Features; ER, Emotional Release; RI, Role Immersion; FI, Fragmented Information; PAR, Precision Algorithmic Recommendation; AD, Attention Deprivation; HP, Hedonic Pleasure; LC, Loss of Control; SVA, Short-Video Addiction; EF, Emotional Fatigue; TD, Time Distortion; SA, Social Avoidance; RSA, Reality Social Avoidance; CF, Cognitive Fatigue.

In indirect relationship and chain mediation, HP plays an important role in SP (IF, ER and RI play a mediating role in the influence of SVA). LC in PP (FI, PAR and AD) mediates the effects of SVA. SVA plays a mediating role in the effects of HP on CF and LC on CF. HP and SVA in SP (IF, ER and RI play a chain mediating role in the influence of CF). LC and SVA in PP (FI, PAR and AD) play a chain mediating role in the influence of CF (Table 2).

**Table 2:** Results of indirect effects and sequential mediation analysis.

H	Construct	Path	*β*	*t*	*p*	Decision
H6	SP (IF)	IF → HP → SVA → CF	0.016	2.941	0.003	Supported
	SP (ER)	ER → HP → SVA → CF	0.01	2.464	0.014	Supported
	SP (RI)	RI → HP → SVA → CF	0.012	2.675	0.007	Supported
H7	PP (FI)	FI → LC → SVA → CF	0.013	2.868	0.004	Supported
	PP (PAR)	PAR → LC → SVA → CF	0.018	2.918	0.004	Supported
	PP (AD)	AD → LC → SVA → CF	0.016	2.855	0.004	Supported

We used standardized root mean square residual (SRMR) to assess the average size of the difference between the observed and the expected correlation matrices, which belongs to the absolute goodness-of-fit indices and is acceptable according to the criteria of Hu and Bentler (1998). SRMR < 0.1 is acceptable, and the SRMR in this study is 0.09, indicating a satisfactory model fit.

### 5.2 Asymmetric analysis

The asymmetric method fsQCA was employed to examine how different combinations (“recipes”) of antecedent conditions is linked to CF. This approach complements PLS-SEM by capturing nonlinear interactions and causal complexity among variables.

Based on causal complexity theory and the principle of parsimony, the analysis focuses on the core antecedent variables: social presence (SP: IF, ER, RI) and physical presence (PP: FI, PAR, AD). The analysis follows three steps: data calibration, necessity analysis, and sufficiency analysis.

In the data calibration process, raw data were transformed into fuzzy-set membership scores using three qualitative anchors: full membership, crossover point, and full non-membership (Ragin, 2008). Following common practice in fsQCA studies using Likert-scale data (Pappas & Woodside, 2021), the thresholds were set at 4 (full membership), 3 (crossover point), and 2 (full non-membership), representing high, neutral, and low levels of construct presence.

Considering the potential clustering around the midpoint (3), the empirical distribution of the data was examined to ensure that the crossover point did not is linked to excessive ambiguity (Fiss, 2011; Rihoux & Ragin, 2009). To avoid ambiguity at the crossover point and ensure that no cases were excluded, a small constant (0.001) was added to membership scores of 0.5. This adjustment preserves cases while maintaining their interpretation near maximum ambiguity (Ragin, 2008). Additional robustness checks using alternative crossover thresholds (2.9 and 3.1) produced substantively similar configurational solutions, indicating that the results were stable and not sensitive to minor calibration adjustments.

#### 5.2.1 Necessity analysis

The necessity analysis examines whether any single antecedent condition is required for the occurrence of CF. Following established criteria (Pappas, 2018), a condition is considered necessary if its consistency exceeds 0.90.

The results indicate that none of the examined conditions (IF, ER, RI, FI, PAR, AD) meets this threshold, suggesting that no single factor is necessary for explaining CF. (see Supplementary Table 5 Analysis of necessary conditions for Cognitive Fatigue).

#### 5.2.2 Analysis of sufficient conditions

Table 3 presents the sufficient configurations derived from the truth table analysis.

**Table 3:** Sufficient recipes to predict high CF.

Configuration	Solution			
	p1	p2	p3	p4
IF	●	●	●	●
ER	●	●	●	⊗
RI	●	●	⊗	●
FI	•	⊗	•	•
PAR	⊗	⊗	•	•
AD	⊗	•	•	•
Raw coverage	0.650562	0.662158	0.605523	0.602532
Unique coverage	0.0506974	0.0622934	0.0463189	0.0433279
Consistency	0.89581	0.891528	0.892385	0.89912
Solution coverage	0.802502			
Solution consistency	0.873436			

Big “●” indicates the presence, Small “•” and”⊗” indicates the negation of an antecedent condition.

The truth table was refined based on frequency and consistency thresholds (Ragin, 2008). Following Fiss (2011), a frequency threshold of eight was applied given the sample size (*N* = 649), and configurations with consistency below 0.80 were excluded (Greckhamer et al., 2013). The intermediate solution was selected for interpretation as it balances theoretical guidance and empirical evidence.

The results identify four configurations leading to high CF. The solution coverage (0.8035) indicates that these configurations collectively explain 80.35% of CF cases, while the solution consistency (0.8731) demonstrates strong predictive reliability.

Specifically:

- Configuration 1: High IF, ER, FI, and RI → high CF
- Configuration 2: High IF, ER, RI, and AD → high CF
- Configuration 3: High IF, ER, FI, PAR, and AD → high CF
- Configuration 4: High IF, FI, RI, PAR, and AD → high CF

These findings suggest that CF is driven by multiple configurational pathways rather than a single dominant factor.

## 6 Discussion

This study developed a pathway-based theoretical model to explain how presence-related experiences contribute to cognitive fatigue (CF) in Douyin short-video viewing environments. The findings support the proposed hedonic and control-loss pathways and further demonstrate the sequential psychological mechanisms through which social presence (SP) and physical presence (PP) indirectly influence CF.

### 6.1 Research objective 1: Core pathways linking presence, addiction, and cognitive fatigue

This study addresses RO1 through the two core pathways proposed in the conceptual model: the hedonic pathway (H1–H2) and the control-loss pathway (H3–H4), both of which explain how SP and PP contribute to SVA. The cognitive consequence of SVA on CF is further examined in H5.

Results support H1 and H2, indicating that SP significantly enhances HP, which subsequently strengthens SVA. Specifically, IF, ER, and RI collectively contribute to pleasurable and immersive experiences that reinforce repeated platform engagement. These findings align with Zhang et al. (2022), Zhou et al. (2023), and Siles et al. (2024), suggesting that immersive social interaction and emotional engagement increase users’ psychological involvement and gratification in short-video environments. ER further promotes psychological safety and emotional satisfaction, echoing UGT’s emphasis on emotional gratification as a key determinant of media behavior (Pang, 2021). This interpretation is additionally supported by Rimé et al. (2020) and Cadet and Chainay (2020), who emphasized the reinforcing effects of emotional sharing, immersion, and presence on users’ affective experiences.

The results also support H3 and H4, indicating that PP significantly increases LC, which subsequently strengthens SVA. FI, PAR, and AD reduce users’ ability to regulate media consumption by continuously stimulating attention and reinforcing passive viewing behavior. These findings are consistent with Mendelson (2024), Mao et al. (2023), Meng (2021), Qu et al. (2024), and Gilbert et al. (2024), supporting information overload theory and media dependency theory. In algorithm-driven environments, users gradually become dependent on automated recommendations and continuous stimulation, thereby weakening self-regulation and increasing compulsive viewing tendencies.

Furthermore, H5 is supported, showing that SVA significantly increases CF. Persistent and excessive short-video engagement continuously consumes attentional and cognitive resources, reducing users’ recovery capacity and impairing information processing. This finding aligns with Hsu et al. (2024), Kaur et al. (2021), Faraci and Nasonte (2024), Nguyen et al. (2025), Tang et al. (2026), Mona et al. (2026), and Li et al. (2025), suggesting that problematic short-video use intensifies cognitive overload, attentional depletion, emotional exhaustion, and cognitive impairment.

Overall, the findings suggest that CF may not emerge directly from presence itself. Instead, SP appears to contribute to CF through hedonic reinforcement, whereas PP may contribute to CF through reduced self-regulation and loss of control. These results support the mechanism-oriented framework proposed in this study and are consistent with prior discussions on pathway-based media effects and psychological dependency processes (Thomas, 2022).

### 6.2 Research objective 2: Sequential mediation mechanisms

RO2 is addressed through the sequential mediation hypotheses (H6–H7), which explain the underlying psychological mechanisms linking presence to cognitive fatigue.

The findings support H6, demonstrating that HP and SVA jointly mediate the relationship between SP and CF. Specifically, IF, ER, and RI indirectly contribute to CF through increased hedonic engagement and addictive viewing behavior. These findings are consistent with Jiang and Yoo (2024), Xu et al. (2024), Zhou et al. (2023), and Siles et al. (2024), indicating that pleasurable and emotionally immersive experiences encourage repeated platform use and strengthen dependence on short-video consumption. Over time, sustained hedonic reinforcement increases compulsive engagement and cognitive burden, ultimately contributing to CF. These findings are further supported by recent evidence syntheses showing that problematic short-video use is closely associated with attentional depletion, emotional exhaustion, and cognitive impairment (Nguyen et al., 2025; Tang et al., 2026).

Similarly, H7 is supported, indicating that LC and SVA jointly mediate the relationship between PP and CF. FI, PAR, and AD indirectly contribute to CF by weakening users’ self-regulation and increasing compulsive viewing behavior. These findings support the argument that algorithmic recommendation systems and fragmented information environments gradually reduce users’ cognitive autonomy and intensify platform dependence (Meng, 2021; Miguel et al., 2024; Qu et al., 2024). This interpretation is also consistent with Gilbert et al. (2024), who demonstrated that immersive digital environments can impair disengagement ability and weaken self-control processes. As exposure accumulates, users become increasingly immersed in passive and repetitive consumption patterns, thereby exacerbating cognitive overload and fatigue.

Taken together, these findings suggest that SP and PP influence CF indirectly through sequential psychological and behavioral mechanisms rather than through direct effects. The results therefore support the theoretical argument that addiction-related processes are central to understanding the cognitive consequences of short-video use and reinforce pathway-based interpretations of media effects mechanisms (Thomas, 2022).

### 6.3 Research objective 3: Moderating effects

RO3 is addressed through the moderation hypothesis (H8), which examines whether EF, TD, and RSA condition the relationship between SVA and CF.

However, the moderating effects of EF, TD, and RSA were not supported. Rather than suggesting that these variables are unimportant, this finding indicates that their roles in short-video contexts may be more complex than initially theorized.

One possible interpretation is that EF and TD may operate as downstream responses or mediating mechanisms rather than moderators in short-video environments. Prior studies have suggested that emotional exhaustion and disengagement difficulties often emerge as consequences of sustained digital media exposure and compulsive usage processes rather than as stable boundary conditions (Sheng et al., 2023; Gilbert et al., 2024). Although post-hoc mediation analyses were beyond the scope of the current study, future research may further examine whether EF and TD function as mediating or outcome variables within the proposed framework. In algorithm-driven environments, users often continue consuming content despite experiencing fatigue or distorted time perception due to ongoing hedonic engagement and reduced self-control (Jiang and Yoo, 2024; Qu et al., 2024). As such, EF and TD may reflect consequences of sustained usage rather than conditions that systematically alter the relationship between SVA and CF.

Additionally, the relationship between SVA and CF appears to be relatively direct and primarily driven by cognitive overload and attentional depletion. Prior studies have shown that excessive digital media engagement continuously consumes attentional resources, impairs concentration, and increases cognitive burden (Rosen et al., 2013; Chen et al., 2023). This may reduce the extent to which contextual or psychological factors such as EF, TD, and RSA exert moderating effects.

Although some individual effects in the model are modest in magnitude, this does not necessarily imply limited practical significance. In complex behavioral systems such as short-video use, cognitive fatigue is more likely to emerge from cumulative and sequential processes rather than single dominant factors. In this study, presence influences CF indirectly through HP, LC, and SVA, forming a multi-stage mechanism. While each individual link may be relatively small, their combined effects may accumulate over time and become practically meaningful in real-world usage contexts. This is particularly relevant in habitual media consumption contexts, where repeated exposure may gradually accumulate cognitive burden over time. This interpretation is consistent with process-oriented perspectives on media effects, which emphasize that psychological outcomes often emerge through accumulative and interacting mechanisms rather than isolated direct relationships (Thomas, 2022).

These findings suggest that future research may benefit from re-examining the roles of EF and TD within the theoretical framework, for example by exploring their potential positions as mediating or outcome variables using longitudinal or experimental designs. Given the cross-sectional design of this study, the findings should be interpreted as associative rather than strictly causal relationships. Future longitudinal and experimental studies are needed to further validate the proposed mechanisms and clarify the temporal dynamics underlying short-video addiction and cognitive fatigue.

### 6.4 Research objective 4: Configurational pathways leading to cognitive fatigue

Fourth, regarding Research Objective 4, the fsQCA results reveal that no single pathway independently leads to CF; instead, multiple configurations jointly contribute to cognitive fatigue. This finding supports the configurational perspective that complex psychological outcomes may emerge through different combinations of interacting conditions rather than through one dominant causal factor (Fiss, 2011).

The interplay among SP, PP, and psychological mechanisms suggests that balancing PAR–FI and ER–AD relationships is particularly important, reflecting the multifactor and nonlinear nature of CF in short-video environments. All four configurations include IF, indicating that immersive flow-related experiences consistently contribute to CF by simultaneously enhancing social engagement and increasing attentional demands (Chen et al., 2023; Xu et al., 2024). ER also appears across all configurations, suggesting its dual role in digital media environments. While emotional resonance may temporarily relieve stress and enhance engagement, excessive emotional stimulation may intensify cognitive burden, particularly when combined with fragmented information exposure or compulsive viewing tendencies (Rimé et al., 2020; Sheng et al., 2023). RI is similarly present in all configurations, indicating that deep psychological involvement in short-video environments may reduce sensitivity to external cognitive cues and contribute to mental fatigue over time.

AD appears in Configuration 2 but not Configuration 1, suggesting that algorithm-driven recommendation mechanisms may directly intensify repetitive exposure and passive consumption patterns, thereby increasing cognitive burden and attentional dependency (Meng, 2021; Siles et al., 2024). PAR is featured in Configuration 3 but absent in Configuration 4, implying that personalized filtering mechanisms may facilitate selective exposure while simultaneously reducing users’ active cognitive processing. When FI and RI co-occur, they appear to further amplify attentional fragmentation and cognitive overload. Overall, the findings suggest that PAR may structure information exposure, whereas FI increases fragmented attention; when both coexist, cognitive tension and mental fatigue become more pronounced.

The fsQCA findings complement the SEM results by demonstrating that cognitive fatigue does not arise from a single dominant factor, but from multiple interacting configurations of SP, PP, and psychological mechanisms. While SEM identifies HP and LC as key mediating pathways, fsQCA further reveals that different combinations of IF, ER, RI, FI, PAR, and AD can jointly produce high CF. This highlights the configurational, nonlinear, and asymmetric nature of short-video effects, extending the linear explanations provided by SEM and reinforcing the value of combining symmetric and asymmetric analytical approaches in digital media research (Fiss, 2011; Hair et al., 2017).

## 7 Conclusion

### 7.1 Theoretical implications

This study extends short-video research by integrating SP and PP into a unified framework for understanding CF in algorithm-driven media environments. The findings suggest that immersive and algorithmically structured viewing experiences are associated with both hedonic engagement and cognitive burden in short-video use contexts.

By distinguishing SP (interaction features, emotional release, and role immersion) from PP (fragmented information, precision algorithmic recommendation, and attention deprivation), the study advances presence theory beyond traditional spatial immersion contexts and adapts it to algorithmically mediated short-video environments. In particular, the study conceptualizes PP as a form of perceptual immersion emerging from sustained attentional engagement and continuous content exposure rather than spatial simulation.

The study further clarifies how presence-related experiences may gradually evolve into maladaptive psychological and cognitive outcomes through sequential mechanisms involving HP, LC, and SVA. Rather than emphasizing isolated direct effects, the findings support a pathway-based interpretation in which cognitive fatigue emerges through cumulative and interacting psychological processes.

Methodologically, the combination of PLS-SEM and fsQCA provides complementary insights into both symmetrical relationships and configurational patterns associated with CF. While PLS-SEM identifies key sequential associations among psychological mechanisms, fsQCA further demonstrates that multiple combinations of SP- and PP-related conditions may jointly correspond to high cognitive fatigue. This combined analytical approach contributes to a more nuanced understanding of nonlinear and configurational dynamics in digital media research.

Although the moderating effects of EF, TD, and RSA were not supported, their inclusion contributes to ongoing discussions regarding the complex psychological mechanisms associated with short-video use.

Overall, the integration of presence theory, uses and gratifications theory, media dependence theory, and information overload theory provides a theoretically integrated framework for understanding the psychological and cognitive implications of algorithm-driven short-video consumption.

### 7.2 Practical implications

The findings offer actionable insights for designers, practitioners, and policymakers. Platform designers should promote algorithmic transparency, introduce screen-time reminders, and implement age-based content controls to reduce over-immersion. For healthcare professionals, understanding HP and LC enables targeted interventions and digital detox strategies. Educators and parents can apply these findings to strengthen digital literacy and attention management, especially among youth. At the policy level, regulatory bodies should enforce algorithm accountability, screen-time limits for minors, and mental health support integrated into digital platforms. Cross-platform cooperation and data sharing can help prevent migratory addictive behavior. Collectively, these measures can mitigate the cognitive and psychological harms of excessive short-video use and foster a healthier, more resilient digital ecosystem.

### 7.3 Limitations and future work

Several limitations provide directions for future research. First, this study focused on Chinese Douyin users aged 18–35, which may limit the external validity and generalizability of the findings across cultures, age groups, and social media platforms. Adolescents under 18, who may be particularly vulnerable to short-video addiction and cognitive fatigue due to developing cognitive control and emotional regulation systems, were excluded due to ethical considerations. Therefore, the observed relationships may not fully capture the intensity of addictive behaviors among younger users. In addition, the findings should be interpreted within the context of Chinese digital culture and the platform-specific characteristics of Douyin, including algorithmic recommendation mechanisms, intensive mobile media usage, and highly interactive short-video ecosystems. Since platforms such as TikTok, Instagram Reels, and YouTube Shorts differ in content formats, interaction mechanisms, and recommendation systems, future studies should conduct cross-cultural and cross-platform comparisons involving adolescent and multi-age samples.

Second, the use of cross-sectional self-reported data limits causal inference and may introduce potential common method bias. Although Harman’s single-factor test and full collinearity VIF assessments indicated that common method bias was unlikely to seriously threaten the findings, the reliance on single-source self-reported data may still introduce potential method-related variance, including social desirability and response consistency biases. Moreover, VIF-based and Harman’s single-factor diagnostics provide only indirect assessments and cannot completely eliminate common method bias concerns. Future studies are encouraged to adopt longitudinal, experimental, or multi-source designs and incorporate objective behavioral tracking data, such as screen-time records or platform usage logs, to strengthen causal inference and reduce potential method bias.

Third, although this study examined hedonic pleasure and loss of control as key psychological mechanisms, other emotional and psychological factors, such as anxiety, loneliness, emotion regulation, self-control, and emotional intelligence, may also influence addictive behaviors and cognitive fatigue. In addition, the non-significant moderating effects observed for several variables suggest that more refined conceptualization and measurement approaches may be needed in future research. Overall, this study provides a foundation for further exploration of presence-driven psychological outcomes in algorithmic media environments.

## Data Availability

The original contributions presented in the study are included in the article/supplementary material, further inquiries can be directed to the corresponding author/s.

## References

[ref1] Akbari M. H. Pishghadam R. (2022). Developing new software to analyze the emo-sensory load of language. J. Bus. Commun. Technol. 1, 1–13. doi: 10.56632/bct.2022.1101

[ref2] Bagger C. (2024). Connectivity as productivity: workplace from Meta and organizational datafication. Media Cult. Soc. 47:1270941. doi: 10.1177/01634437241270941

[ref3] Cadet L. B. Chainay H. (2020). Memory of virtual experiences: role of immersion, emotion and sense of presence. Int. J. Hum. Comput. Stud. 144:102506. doi: 10.1016/j.ijhcs.2020.102506

[ref9020] Campbell S. Greenwood M. Prior S. Shearer T. Walkem K. Young S. . (2020). Purposive sampling: complex or simple? Research case examples. J. Res. Nurs. 25, 652–661. doi: 10.1177/1744987120927206 34394687 PMC7932468

[ref9021] Cohen J. (1988). Statistical Power Analysis for the Behavioral Sciences. 2nd Edn. Hillsdale, NJ: Lawrence Erlbaum Associates.

[ref4] Chen Y. Li M. Guo F. Wang X. (2023). The effect of short-form video addiction on users’ attention. Behav. Inf. Technol. 42, 2893–2910. doi: 10.1080/0144929X.2022.2151512

[ref5] Daassi M. Debbabi S. (2021). Intention to reuse AR-based apps: the combined role of the sense of immersion, product presence and perceived realism. Inf. Manag. 58:103453. doi: 10.1016/j.im.2021.103453

[ref9016] Dul J. (2016). Identifying single necessary conditions with NCA and fsQCA. J. Bus. Res. 69, 1516–1523. doi: 10.1016/j.jbusres.2015.10.134

[ref6] Egliston B. Carter M. (2024). ‘The metaverse and how we’ll build it’: the political economy of Meta’s reality labs. New Media Soc. 26, 4336–4360. doi: 10.1177/14614448221119785

[ref9022] Erdfelder E. Faul F. Buchner A. Lang A.-G. (2009). Statistical power analyses using G*power 3.1: tests for correlation and regression analyses. *Behav. Res.* Methods 41, 1149–1160. doi: 10.3758/BRM.41.4.1149 19897823

[ref7] Faraci P. Nasonte G. (2024). The brief social media fatigue scale (BSMFS): a new short version through exploratory structural equation modeling and associations with trait anxiety, fear of missing out, boredom proneness, and problematic use. Int. J. Hum. Comput. Interact. 1, 1–27.

[ref9023] Faul F. Erdfelder E. Lang A.-G. Buchner A. (2007). G*power 3: a flexible statistical power analysis program for the social, behavioral, and biomedical sciences. Behav. Res. Methods 39, 175–191. doi: 10.3758/BF03193146 17695343

[ref8] Fiss P. C. (2011). Building better causal theories: a fuzzy set approach to typologies in organisation research. Acad. Manag. J. 54, 393–420. doi: 10.5465/AMJ.2011.60263120

[ref9015] Falk R. F. Miller N. B. (1992). A Primer for Soft Modeling. Akron, OH: University of Akron Press.

[ref9014] Fontes-Perryman E. Spina R. (2022). Fear of missing out and compulsive social media use as mediators between OCD symptoms and social media fatigue. *Psychol. Pop.* Media 11, 173–181. doi: 10.1037/ppm0000356

[ref10] Gilbert A. Reinecke L. Meier A. Baumgartner S. E. Dietrich F. (2024). Too amused to stop? Self-control and the disengagement process on Netflix. J. Commun. 74, 387–398. doi: 10.1093/joc/jqae023

[ref9013] Grall C. Tamborini R. Weber R. Schmälzle R. (2021). Stories collectively engage listeners’ brains: enhanced intersubject correlations during reception of personal narratives. J. Commun. 71, 332–355. doi: 10.1093/joc/jqab004

[ref9025] Greckhamer T. Misangyi V. F. Elms H. Lacey R. (2013). Using qualitative comparative analysis in strategic management research: an examination of combinations of industry, corporate, and business-unit effects. *Organ. Res.* Methods 16, 463–487. doi: 10.1177/1094428107302907

[ref9012] Hair J. F. Hult G. T. M. Ringle C. M. Sarstedt M. (2016). A Primer on Partial Least Squares Structural Equation Modeling (PLS-SEM). 2nd Edn. Thousand Oaks, CA: Sage Publications.

[ref11] Hair J. F. Matthews L. M. Matthews R. L. Sarstedt M. (2017). PLS-SEM or CB-sem: updated guidelines on which method to use. Int. J. Multivariate Data Anal. 1, 107–123. doi: 10.1504/IJMDA.2017.087624

[ref13] Hsu J. S. C. Chiu C. M. Chang-Chien Y. T. Tang K. (2024). How social media fatigue feigning and altering emotion discourage the use of social media. Internet Res. 34, 1488–1518. doi: 10.1108/INTR-06-2022-0390

[ref9024] Hu L. T. Bentler P. M. (1998). Fit indices in covariance structure modeling: sensitivity to underparameterized model misspecification. Psychol. Methods 3, 424–453. doi: 10.1037/1082-989X.3.4.424

[ref14] Irom B. (2022). Virtual reality and celebrity humanitarianism: Rashida Jones in Lebanon. Media Cult. Soc. 44, 88–104. doi: 10.1177/01634437211022725

[ref9010] Jackson T. W. Farzaneh P. (2012). Theory-based model of factors affecting information overload. Int. J. Inf. Manag. 32, 523–532. doi: 10.1016/j.ijinfomgt.2012.04.006

[ref9011] Jabeen F. Tandon A. Sithipolvanichgul J. Srivastava S. Dhir A. (2023). Social media-induced fear of missing out (FoMO) and social media fatigue: the role of narcissism, comparison and disclosure. J. Bus. Res. 159:113693. doi: 10.1016/j.jbusres.2023.113693

[ref15] Jiang L. Yoo Y. (2024). Adolescents’ short-form video addiction and sleep quality: the mediating role of social anxiety. BMC Psychol. 12:369. doi: 10.1186/s40359-024-01865-9, 38943173 PMC11214215

[ref9009] Kaye D. B. V. Chen X. Zeng J. (2021). The co-evolution of two Chinese mobile short video apps: parallel platformization of Douyin and TikTok. Mob. *Media Commun.* 9, 229–253. doi: 10.1177/2050157920952120

[ref9026] Kock N. (2015). Common method bias in PLS-SEM: a full collinearity assessment approach. Int. J. e-Collab. 11, 1–10. doi: 10.4018/ijec.201510010

[ref16] Kaur P. Islam N. Tandon A. Dhir A. (2021). Social media users’ online subjective well-being and fatigue: a network heterogeneity perspective. Technol. Forecast. Soc. Change 172:121039. doi: 10.1016/j.techfore.2021.121039

[ref17] Kim J. W. Guess A. Nyhan B. Reifler J. (2021). The distorting prism of social media: how self-selection and exposure to incivility fuel online comment toxicity. J. Commun. 71, 922–946. doi: 10.1093/joc/jqab034

[ref18] Kruzan K. P. Won A. S. (2019). Embodied well-being through two media technologies: virtual reality and social media. New Media Soc. 21, 1734–1749. doi: 10.1177/1461444819829873

[ref19] Li T. Liu H. Hu R. Liu X. (2025). Research insights on ‘problematic use of short video’: a comprehensive review exploration in psychological context. Addict. Biol. 30:e70082. doi: 10.1111/adb.70082, 41194576 PMC12589815

[ref20] Li M. Ma Q. (2024). The effect of social media fatigue on compulsive buying. Behav. Inf. Technol. 44:1429. doi: 10.1080/0144929x.2024.2358359

[ref9008] Liu T. Xu M. Chen X. (2021). Social media, gendered anxiety and disease-related misinformation: discourses in contemporary China’s online anti-African sentiments. Asian J. Commun. 31, 485–501. doi: 10.1080/01292986.2021.1941150

[ref9007] Mao Z. Guan Z. Gu X. (2023). How do level of novelty and camera angle of tourism-themed short videos on Douyin influence potential travelers’ behavioral intentions? Cyberpsychol. Behav. Soc. Netw. 26, 672–678. doi: 10.1089/cyber.2022.0108 37639706

[ref22] Meng J. (2021). Discursive contestations of algorithms: a case study of recommendation platforms in China. Chin. J. Commun. 14, 313–328. doi: 10.1080/17544750.2021.1875491

[ref9006] Mendelson E. A. (2024). Sensemaking and public intimacy on TikTok: how viral videos influence interpersonal relationships offline. New Media Soc. 26, 7081–7099. doi: 10.1177/14614448231163

[ref23] Miguel C. Lutz C. Perez-Vega R. Majetić F. (2024). “Alone on the road”: loneliness among digital nomads and the use of social media to foster personal relationships. Media Cult. Soc. 47:290087. doi: 10.1177/01634437241290087

[ref24] Mona A. E. Roshith V. Peter R. Roy P. Hassan A. Devika M. . (2026). Short video addiction and its impact on cognitive functioning in adolescents and youth: a systematic review. Int. J. Adolesc. Youth 31:2623337. doi: 10.1080/02673843.2026.2623337

[ref25] Nguyen L. Walters J. Paul S. Monreal Ijurco S. Rainey G. E. Parekh N. . (2025). Feeds, feelings, and focus: a systematic review and meta-analysis examining the cognitive and mental health correlates of short-form video use. Psychol. Bull. 151, 1125–1146. doi: 10.1037/bul0000498, 41231585

[ref9005] Ning C. Miao R. Wang T. (2024). Communicating via gold medal: Chinese Olympic athletes’ visual self-presentation on the social media platform Douyin. Chin. J. Commun. doi: 10.1080/17544750.2024.2310523

[ref26] Pang H. (2021). Identifying associations between mobile social media users’ perceived values, attitude, satisfaction, and eWOM engagement: the moderating role of affective factors. Telemat. Inform. 59:101561. doi: 10.1016/j.tele.2020.101561

[ref9027] Pappas I. O. (2018). User experience in personalized online shopping: a fuzzy-set analysis. Eur. J. Mark. 52, 1679–1703. doi: 10.1108/EJM-10-2017-0707

[ref9019] Pappas I. O. Woodside A. G. (2021). Fuzzy-set qualitative comparative analysis (fsQCA): guidelines for research practice in information systems and marketing. Int. J. Inf. Manag. 58:102310. doi: 10.1016/j.ijinfomgt.2021.102310

[ref9003] Peterson-Salahuddin C. (2024). “Pose”: examining moments of “digital” dark sousveillance on TikTok. New Media Soc. 26, 2387–2406. doi: 10.1177/1461444822108

[ref9004] Pernagallo G. Torrisi B. (2022). A theory of information overload applied to perfectly efficient financial markets. *Rev. Behav.* Finance 14, 223–236. doi: 10.1108/RBF-07-2019-0088

[ref27] Qu D. Liu B. Jia L. Zhang X. Chen D. Zhang Q. . (2024). The longitudinal relationships between short video addiction and depressive symptoms: a cross-lagged panel network analysis. Comput. Hum. Behav. 152:108059. doi: 10.1016/j.chb.2023.108059

[ref9017] Ragin C. C. (2008). Redesigning Social Inquiry: Fuzzy Sets and beyond. Chicago, IL: University of Chicago Press.

[ref28] Reybrouck M. Eerola T. (2022). Musical enjoyment and reward: from hedonic pleasure to eudaimonic listening. Behav. Sci. 12:154. doi: 10.3390/bs12050154, 35621451 PMC9137732

[ref29] Rimé B. Bouchat P. Paquot L. Giglio L. (2020). Intrapersonal, interpersonal, and social outcomes of the social sharing of emotion. Curr. Opin. Psychol. 31, 127–134. doi: 10.1016/j.copsyc.2019.08.024, 31590107

[ref9018] Rihoux B. Ragin C. C. (Eds.) (2009). Configurational Comparative Methods: Qualitative Comparative Analysis (QCA) and Related Techniques. Thousand Oaks, CA: Sage Publications.

[ref30] Rosen L. D. Carrier L. M. Cheever N. A. (2013). Facebook and texting made me do it: media-induced task-switching while studying. Comput. Hum. Behav. 29, 948–958. doi: 10.1016/j.chb.2012.12.001

[ref31] Saker M. Frith J. (2020). Coextensive space: virtual reality and the developing relationship between the body, the digital and physical space. Media Cult. Soc. 42, 1427–1442. doi: 10.1177/0163443720932498

[ref32] Sheng N. Yang C. Han L. Jou M. (2023). Too much overload and concerns: antecedents of social media fatigue and the mediating role of emotional exhaustion. Comput. Hum. Behav. 139:107500. doi: 10.1016/j.chb.2022.107500

[ref33] Siles I. Valerio-Alfaro L. Meléndez-Moran A. (2024). Learning to like TikTok. And not: algorithm awareness as process. New Media Soc. 26, 5702–5718. doi: 10.1177/14614448221138973

[ref34] Tang D. Zhang X. Gou P. Feng J. Hu R. Sum K. W. R. (2026). Association between short-form video use and mental health: systematic review and Meta-analysis. J. Med. Internet Res. 28:e82503. doi: 10.2196/82503, 41849573 PMC12998613

[ref35] Thomas F. (2022). A methodological framework for analyzing the appearance and duration of media effects. J. Commun. 72, 401–428. doi: 10.1093/joc/jqac013

[ref36] Wang Y. (2020). Humor and camera view on mobile short-form video apps influence user experience and technology-adoption intent, an example of TikTok (DouYin). Comput. Human Behav. 110:106373. doi: 10.1016/j.chb.2020.106373

[ref9002] Wang C. Lee M. K. O. Hua Z. (2015). A theory of social media dependence: evidence from microblog users. Decis. Support. Syst. 69, 40–49. doi: 10.1016/j.dss.2014.11.002

[ref37] Wang C. Yan J. Huang L. Cao N. (2024). Helping middle-aged and elderly short-video creators attract followers: a mixed-methods study on Douyin users. Inf. Technol. People 37, 1305–1333. doi: 10.1108/ITP-03-2022-0203

[ref38] Weber R. Behr K. M. Fisher J. T. Lonergan C. Quebral C. (2020). Video game violence and interactivity: effect or equivalence? J. Commun. 70, 219–244. doi: 10.1093/joc/jqz048

[ref39] Xu J. Ramayah T. Arshad M. Z. Ismail A. Jamal J. (2024). Decoding digital dependency: flow experience and social belonging in short video addiction among middle-aged and elderly Chinese users. Telemat. Inform.:102222. doi: 10.1016/j.tele.2024.102222

[ref40] Yildiz H. Reiter A. Vrontis D. Mouline J. P. (2024). Interacting in virtual reality: when the Proteus effect stimulates 3D MMORPG players to buy. Technol. Forecast. Soc. Change 201:123205. doi: 10.1016/j.techfore.2024.123205

[ref9001] Yu Z. (2021). An empirical study of consumer video activism in China: protesting against businesses with short videos. Chin. J. Commun. 14, 297–312. doi: 10.1080/17544750.2020.1871390

[ref41] Zhang Y. Bu R. Li X. (2024). Social exclusion and short video addiction: the mediating role of boredom and self-control. Psychol. Res. Behav. Manag. 17, 2195–2203. doi: 10.2147/PRBM.S463240, 38832345 PMC11146338

[ref42] Zhang W. Chen Z. Xi Y. (2020). Traffic media: how algorithmic imaginations and practices change content production. Chin. J. Commun. 14, 58–74. doi: 10.1080/17544750.2020.1830422, 37339054

[ref43] Zhang Y. He W. Peng L. (2022). How perceived pressure affects users’ social media fatigue behaviour: a case on WeChat. J. Comput. Inf. Syst. 62, 337–348. doi: 10.1080/08874417.2020.1824596

[ref44] Zhou F. Lin Y. Mou J. Cohen J. Chen S. (2023). Understanding the dark side of gamified interactions on short-form video platforms: through a lens of expectations violations theory. Technol. Forecast. Soc. Change 186:122150. doi: 10.1016/j.techfore.2022.122150

