# Supplementary material for: The dark side of algorithmic entertainment: social and physical presence, short video addiction, and cognitive fatigue among Douyin users
Source: Front Psychol. 2026 Jun 15;17:1856148. doi: 10.3389/fpsyg.2026.1856148 (PMC13312163; doi:10.3389/fpsyg.2026.1856148)
Supplement: Supplementary file 1 [file Table_1.docx]

**Table 1**

Constructs, items, and their sources

| **Variable Name** | **Items** | **Variables Reference** |
| --- | --- | --- |
| Interaction Features | IF1:I will frequently use the like, comment, or share features of short video platforms. | Zhang,et al., (2019); Kim et al., (2021) ; Siles et al., (2024) |
|  | IF2:I interact with other users on short video platforms very frequently (e.g., comment interaction). |  |
|  | IF3:I like to express my opinions and emotions through the interactive features of short video platforms. |  |
| Emotional Release | ER1:I release stress and negative emotions by watching short videos. | Rubin, (2002); Rimé et al., (2020); Reybrouck & Eerola, (2022) |
|  | ER2:Watching short videos makes me feel relaxed when I feel anxious or depressed. |  |
|  | ER3:Short videos help me to express and regulate my emotions. |  |
|  | ER4:I enjoy getting emotional catharsis and release through short video content. |  |
| Role Immersion | RI1:When watching short videos, I often feel like I am in the situation or character presented in the video. | Bodzin et al., (2021); Cadet & Chainay, (2020); Han et al., (2023) |
|  | RI2:I can deeply experience the emotions of the characters and the storyline of the video. |  |
|  | RI3:I forget about the real world for a while and become completely immersed when watching short videos. |  |
| Fragmented Information | FI1:I often receive fragmented and incomplete information on short videos. | Yin et al., (2024); Zhang et al., (2020); Meng, (2021) |
|  | FI2:The information in short videos is usually disjointed and requires me to watch multiple times to fully understand it. |  |
|  | FI3:I feel that most of the information on short video platforms is fragmented and lacks depth. |  |
|  | FI4:I often encounter information on short video platforms that is broken up into multiple parts and it takes me time to piece together the complete content. |  |
| Precision Algorithmic Recommendation | PAR1:I think the recommendation algorithms on short video platforms are able to accurately push content that interests me. | Xu et al., (2024); Siles et al., (2024); Zhou et al., (2023) |
|  | PAR2:I often find that the recommended content on short video platforms is highly aligned with my preferences. |  |
|  | PAR3:The recommendation system of the video platform understands my viewing habits accurately and provides relevant content. |  |
| Attention Deprivation | AD1:I find it difficult to concentrate when using short-form video platforms. | Rosen et al., (2023); Chen et al., (2023); Nguyen et al., (2025) |
|  | AD2:I often feel like I can't fully focus on the content when watching short videos. |  |
|  | AD3:I am often distracted by multiple messages when using short-form video platforms, which makes it impossible for me to concentrate. |  |
| Hedonic Pleasure | HP1:I feel very pleasant and relaxed when watching short videos. | Pang, (2021); Reybrouck & Eerola, (2022) |
|  | HP2:Short videos allow me to experience pleasant emotions and hedonic feelings. |  |
|  | HP3:I find watching short videos to be an enjoyable and entertaining activity. |  |
|  | HP4:I feel a sense of satisfaction and pleasure whenever I watch short videos. |  |
|  | HP5:I often have fun and enjoyment from watching short videos. |  |
| Loss of Control | LC1:When I use short video platforms, I sometimes feel that I have no control over what I watch. | Elhai et al., (2018); Gilbert et al., (2024) |
|  | LC2:The recommendation system of the short video platform makes me feel like I have no control over what I see. |  |
|  | LC3:I feel that when using short videos, the platform's content recommendations often make me lose control of what I want to watch. |  |
| Short-Video Addiction | SVA1:I often spend a lot of time watching short videos, even though I know I should be doing other things. | Tian et al., (2023); Qu et al., (2024); Tang et al., (2026) |
|  | SVA2:Whenever I have free time, I can't help but open the short video platform. |  |
|  | SVA3:I find it hard to stop watching short videos even though I know it affects my daily life. |  |
|  | SVA4:I often feel that I am addicted to the use of short videos and I cannot control the time and frequency of viewing. |  |
| Emotional Fatigue | EF1:I often feel emotionally exhausted while watching short videos. | Sheng et al., (2023); Kaur et al., (2021) |
|  | EF2:Prolonged use of short video platforms makes me feel somewhat emotionally drained. |  |
|  | EF3:Whenever I am immersed in short video content for an extended period of time, I feel emotionally exhausted and a sense of emptiness. |  |
| Time Distortion | TD1:I often forget the passage of time while watching short videos. | Yang et al., (2024); Gilbert et al., (2024); Xu et al., (2024) |
|  | TD2:Whenever I watch short videos, I always feel that time passes exceptionally fast. |  |
|  | TD3:I lose my sense of time when I am immersed in short video content. |  |
|  | TD4:I sometimes spend more time than planned on short video platforms because I don't feel that time passes that fast. |  |
| Reality Social Avoidance | RSA1:I prefer to avoid people in real life instead of engaging in social activities. | Vaux, (1988); Miguel et al., (2024); Jiang & Yoo, (2024) |
|  | RSA2:I often feel anxious in real life so I avoid interacting with others. |  |
|  | RSA3:I feel constantly uncomfortable in real life social situations, so I will try to avoid them. |  |
|  | RSA4:I often feel uncomfortable or avoid others when I have to interact with them face-to-face |  |
| Cognitive Fatigue | CF1:I find my memory gets worse when watching short videos. | Jessen et al., (2020); Chen et al., (2023); Nguyen et al., (2025) |
|  | CF2:Prolonged use of social media platforms makes it difficult for me to concentrate. |  |
|  | CF3:I sometimes have trouble remembering the content of short videos I've watched before, and I feel like my brain is a little sluggish. |  |
|  | CF4:As I spend more time watching short videos, I notice that I have more difficulty thinking about and understanding new information. |  |
