# Supplementary material for: The dark side of algorithmic entertainment: social and physical presence, short video addiction, and cognitive fatigue among Douyin users
Source: Front Psychol. 2026 Jun 15;17:1856148. doi: 10.3389/fpsyg.2026.1856148 (PMC13312163; doi:10.3389/fpsyg.2026.1856148)
Supplement: Supplementary file 2 [file Table_2.docx]

**Table 2**

Measurement model assessment results.

| **Constructs** | **Items** | **Loadings** | **CR** | **AVE** |
| --- | --- | --- | --- | --- |
| Interaction Features | IF1 | 0.843 | 0.891 | 0.732 |
|  | IF2 | 0.864 |  |  |
|  | IF3 | 0.86 |  |  |
| Emotional Release | ER1 | 0.824 | 0.891 | 0.672 |
|  | ER2 | 0.842 |  |  |
|  | ER3 | 0.818 |  |  |
|  | ER4 | 0.793 |  |  |
| Role Immersion | RI1 | 0.872 | 0.892 | 0.734 |
|  | RI2 | 0.847 |  |  |
|  | RI3 | 0.851 |  |  |
| Fragmented Information | FI1 | 0.851 | 0.899 | 0.691 |
|  | FI2 | 0.839 |  |  |
|  | FI3 | 0.805 |  |  |
|  | FI4 | 0.829 |  |  |
| Precision Algorithmic Recommendation | PAR1 | 0.841 | 0.872 | 0.694 |
|  | PAR2 | 0.808 |  |  |
|  | PAR3 | 0.849 |  |  |
| Attention Deprivation | AD1 | 0.841 | 0.882 | 0.714 |
|  | AD2 | 0.853 |  |  |
|  | AD3 | 0.841 |  |  |
| Hedonic Pleasure | HP1 | 0.831 | 0.911 | 0.673 |
|  | HP2 | 0.833 |  |  |
|  | HP3 | 0.821 |  |  |
|  | HP4 | 0.801 |  |  |
|  | HP5 | 0.815 |  |  |
| Loss of Control | LC1 | 0.843 | 0.881 | 0.711 |
|  | LC2 | 0.842 |  |  |
|  | LC3 | 0.844 |  |  |
| Short-Video Addiction | SVA1 | 0.82 | 0.899 | 0.689 |
|  | SVA2 | 0.831 |  |  |
|  | SVA3 | 0.839 |  |  |
|  | SVA4 | 0.831 |  |  |
| Emotional Fatigue | EF1 | 0.853 | 0.891 | 0.732 |
|  | EF2 | 0.85 |  |  |
|  | EF3 | 0.863 |  |  |
| Time Distortion | TD1 | 0.824 | 0.894 | 0.679 |
|  | TD2 | 0.811 |  |  |
|  | TD3 | 0.847 |  |  |
|  | TD4 | 0.813 |  |  |
| Reality Social Avoidance | RSA1 | 0.829 | 0.916 | 0.685 |
|  | RSA2 | 0.828 |  |  |
|  | RSA3 | 0.833 |  |  |
|  | RSA4 | 0.82 |  |  |
|  | RSA5 | 0.828 |  |  |
| Cognitive Fatigue | CD1 | 0.832 | 0.896 | 0.684 |
|  | CD2 | 0.81 |  |  |
|  | CD3 | 0.842 |  |  |
|  | CD4 | 0.824 |  |  |
