# Supplementary material for: The dark side of algorithmic entertainment: social and physical presence, short video addiction, and cognitive fatigue among Douyin users
Source: Front Psychol. 2026 Jun 15;17:1856148. doi: 10.3389/fpsyg.2026.1856148 (PMC13312163; doi:10.3389/fpsyg.2026.1856148)
Supplement: Supplementary file 3 [file Table_3.docx]

**Table 3**

VIF Result.

|  | **VIF** |
| --- | --- |
| **AD1** | 1.634 |
| **AD2** | 1.743 |
| **AD3** | 1.774 |
| **CF1** | 1.900 |
| **CF2** | 1.751 |
| **CF3** | 1.990 |
| **CF4** | 1.883 |
| **EF1** | 1.829 |
| **EF2** | 1.802 |
| **EF3** | 1.802 |
| **ER1** | 1.893 |
| **ER2** | 1.797 |
| **ER3** | 1.843 |
| **ER4** | 1.748 |
| **FI1** | 1.955 |
| **FI2** | 1.993 |
| **FI3** | 1.796 |
| **FI4** | 1.945 |
| **HP1** | 2.083 |
| **HP2** | 2.187 |
| **HP3** | 2.031 |
| **HP4** | 1.874 |
| **HP5** | 2.027 |
| **IF1** | 1.689 |
| **IF2** | 1.904 |
| **IF3** | 1.894 |
| **LC1** | 1.649 |
| **LC2** | 1.718 |
| **LC3** | 1.722 |
| **PAR1** | 1.671 |
| **PAR2** | 1.576 |
| **PAR3** | 1.606 |
| **RI1** | 1.876 |
| **RI2** | 1.745 |
| **RI3** | 1.861 |
| **SA1** | 2.091 |
| **SA2** | 2.154 |
| **SA3** | 2.099 |
| **SA4** | 2.026 |
| **SA5** | 2.138 |
| **SVA1** | 1.864 |
| **SVA2** | 1.955 |
| **SVA3** | 1.937 |
| **SVA4** | 1.868 |
| **TD1** | 1.800 |
| **TD2** | 1.832 |
| **TD3** | 2.024 |
| **TD4** | 1.780 |
| **EF x SVA** | 1.000 |
| **TD x SVA** | 1.000 |
| **SA x SVA** | 1.000 |

**Notes -** IF = Interaction Features, ER = Emotional Release, RI = Role Immersion, FI = Fragmented Information, PAR = Precision Algorithmic Recommendation, AD = Attention Deprivation, HP = Hedonic Pleasure, LC = Loss of Control, SVA = Short-Video Addiction, EF = Emotional Fatigue, TD = Time Distortion, SA = Social Avoidance, RSA = Reality Social Avoidance, CF= Cognitive Fatigue.
