# Supplementary material for: The dark side of algorithmic entertainment: social and physical presence, short video addiction, and cognitive fatigue among Douyin users
Source: Front Psychol. 2026 Jun 15;17:1856148. doi: 10.3389/fpsyg.2026.1856148 (PMC13312163; doi:10.3389/fpsyg.2026.1856148)
Supplement: Supplementary file 4 [file Table_4.docx]

**Table 4**

HTMT results.

|  | **AD** | **CF** | **EF** | **ER** | **FI** | **HP** | **IF** | **LC** | **PAR** | **RI** | **RSA** | **SVA** | **TD** | **EF x SVA** | **SA x SVA** | **TD x SVA** |
| --- | --- | --- | --- | --- | --- | --- | --- | --- | --- | --- | --- | --- | --- | --- | --- | --- |
| **AD** |  |  |  |  |  |  |  |  |  |  |  |  |  |  |  |  |
| **CF** | 0.41 |  |  |  |  |  |  |  |  |  |  |  |  |  |  |  |
| **EF** | 0.412 | 0.451 |  |  |  |  |  |  |  |  |  |  |  |  |  |  |
| **ER** | 0.414 | 0.511 | 0.479 |  |  |  |  |  |  |  |  |  |  |  |  |  |
| **FI** | 0.49 | 0.426 | 0.377 | 0.378 |  |  |  |  |  |  |  |  |  |  |  |  |
| **HP** | 0.439 | 0.537 | 0.445 | 0.496 | 0.469 |  |  |  |  |  |  |  |  |  |  |  |
| **IF** | 0.551 | 0.628 | 0.54 | 0.556 | 0.476 | 0.598 |  |  |  |  |  |  |  |  |  |  |
| **LC** | 0.523 | 0.582 | 0.471 | 0.535 | 0.475 | 0.533 | 0.594 |  |  |  |  |  |  |  |  |  |
| **PAR** | 0.518 | 0.519 | 0.415 | 0.425 | 0.498 | 0.462 | 0.54 | 0.549 |  |  |  |  |  |  |  |  |
| **RI** | 0.475 | 0.51 | 0.417 | 0.51 | 0.482 | 0.533 | 0.577 | 0.657 | 0.45 |  |  |  |  |  |  |  |
| **RSA** | 0.45 | 0.49 | 0.443 | 0.506 | 0.362 | 0.488 | 0.544 | 0.481 | 0.429 | 0.432 |  |  |  |  |  |  |
| **SVA** | 0.475 | 0.521 | 0.534 | 0.535 | 0.467 | 0.53 | 0.593 | 0.616 | 0.487 | 0.682 | 0.503 |  |  |  |  |  |
| **TD** | 0.594 | 0.63 | 0.584 | 0.529 | 0.532 | 0.63 | 0.664 | 0.657 | 0.632 | 0.593 | 0.591 | 0.61 |  |  |  |  |
| **EF x SVA** | 0.145 | 0.077 | 0.272 | 0.109 | 0.149 | 0.071 | 0.12 | 0.056 | 0.107 | 0.019 | 0.068 | 0.243 | 0.118 |  |  |  |
| **SA x SVA** | 0.186 | 0.131 | 0.073 | 0.134 | 0.091 | 0.101 | 0.164 | 0.092 | 0.179 | 0.142 | 0.168 | 0.272 | 0.215 | 0.376 |  |  |
| **TD x SVA** | 0.256 | 0.213 | 0.11 | 0.218 | 0.214 | 0.209 | 0.217 | 0.226 | 0.253 | 0.156 | 0.184 | 0.341 | 0.43 | 0.389 | 0.545 |  |

**Notes -** IF = Interaction Features, ER = Emotional Release, RI = Role Immersion, FI = Fragmented Information, PAR = Precision Algorithmic Recommendation, AD = Attention Deprivation, HP = Hedonic Pleasure, LC = Loss of Control, SVA = Short-Video Addiction, EF = Emotional Fatigue, TD = Time Distortion, SA = Social Avoidance, RSA = Reality Social Avoidance, CF= Cognitive Fatigue.
