# Supplementary material for: The dark side of algorithmic entertainment: social and physical presence, short video addiction, and cognitive fatigue among Douyin users
Source: Front Psychol. 2026 Jun 15;17:1856148. doi: 10.3389/fpsyg.2026.1856148 (PMC13312163; doi:10.3389/fpsyg.2026.1856148)
Supplement: Supplementary file 5 [file Table_5.docx]

**Table 5**

Analysis of necessary conditions for Cognitive Fatigue.

|  | Cognitive Fatigue | | ~Cognitive Fatigue | |
| --- | --- | --- | --- | --- |
| **Condition** | **Consistency** | **Coverage** | **Consistency** | **Coverage** |
| IF | 0.897 | 0.848 | 0.524 | 0.203 |
| ~IF | 0.158 | 0.448 | 0.606 | 0.707 |
| ER | 0.882 | 0.806 | 0.638 | 0.237 |
| ~ER | 0.168 | 0.533 | 0.479 | 0.621 |
| FI | 0.866 | 0.806 | 0.636 | 0.240 |
| ~FI | 0.187 | 0.556 | 0.493 | 0.601 |
| RI | 0.854 | 0.817 | 0.587 | 0.229 |
| ~RI | 0.193 | 0.532 | 0.527 | 0.598 |
| PAR | 0.874 | 0.818 | 0.605 | 0.231 |
| ~PAR | 0.176 | 0.526 | 0.524 | 0.629 |
| AD | 0.864 | 0.785 | 0.703 | 0.258 |
| ~AD | 0.186 | 0.604 | 0.422 | 0.560 |

**Notes -** IF = Interaction Features, ER = Emotional Release, RI = Role Immersion, FI = Fragmented Information, PAR = Precision Algorithmic Recommendation, AD = Attention Deprivation, HP = Hedonic Pleasure, LC = Loss of Control, SVA = Short-Video Addiction, EF = Emotional Fatigue, TD = Time Distortion, SA = Social Avoidance, RSA = Reality Social Avoidance, CF= Cognitive Fatigue.
